# Relationship between mir-126 expression in children with psoriasis, disease progression and therapeutic response

**DOI:** 10.25122/jml-2021-0115

**Published:** 2021

**Authors:** Elvina Murzina, Victor Dosenko, Tetiana Drevytska, Oleksandr Litus, Kateryna Bardova, Svitlana Vozianova

**Affiliations:** 1. Department of Dermatovenereology, Allergology, Clinical and Laboratory Immunology, Shupyk National Healthcare University of Ukraine, Kyiv, Ukraine; 2. Department of General and Molecular Pathophysiology of Bogomoletz Institute of Physiology NAS of Ukraine, Kyiv, Ukraine

**Keywords:** miR-126 expression, children with psoriasis, psoriatic keratinocytes

## Abstract

This study aimed to investigate the expression level of miR-126 in children with psoriasis in the epidermis affected by psoriasis and intact buccal epithelium, establish the impact on the characteristics of the course of psoriasis and the results of therapy in children with psoriasis of initial expression levels of miR-126. miR-126 expression levels in psoriatic keratinocytes and buccal epithelium were determined in 54 children with psoriasis on the severity of psoriasis, treatment efficacy. miR-126 levels in the buccal epithelium in children with psoriasis were reduced compared to healthy children (AUC=0.776±0.048, p<0.001). There were no discrepancies between miR-126 expression levels in psoriatic keratinocytes and buccal epithelium (p=0.097). There are statistically significant discrepancies between miR-126 expression levels in the psoriatic epidermis depending on the clinical form of psoriasis (AUC=0.637±0.056; p=0.014) and severity according to BSA (AUC=0.634±0.063; p=0.034). Depending on the miR-126 level in the buccal epithelium, the response to treatment (PASI<75) in children with high miR-126 is worse than in children with expected miR-126 levels (OR 2.79; 95%; CI: 1.19 – 6.51). Treatment failures were observed in children with high levels of miR-126 in the buccal epithelium compared to miR-126 in the psoriatic epidermis: children aged 12/13 to 17 years (OR 2.44; 95% CI: 1.02 – 5.85), children with PGA=4 (OR 3.16; 95% CI: 1.34 – 7.43). The location and level of miR-126 expression affects the course of psoriasis and the outcome of treatment. High levels of miR-126 in psoriatic keratinocytes lead to manifestations of plaque psoriasis with a course of moderate to severe forms. Initial miR-126 levels in the buccal epithelium in children with psoriasis are a prognostic criterion for response to therapy and can be used as a marker for prescribing systemic treatment.

## Introduction

MicroRNAs (miRNAs) are small non-coding 18–25 nucleotide-long RNA molecules involved in transcriptional and post-transcriptional regulation of gene expression by RNA interference [1–3]. MicroRNAs suppress gene activity, complementary bind to regions of messenger RNA (mRNA), and inhibit its translation. The first microRNAs were described in the early 1990s [4], but they began to be considered as a separate class of biological regulatory molecules with specific functions only in the early 2000s. To date, more than 1,800 human microRNAs are known [5]. Different cells and tissues synthesize different sets of microRNAs, so research leads to the discovery of new molecules every year [6]. According to various estimates, miRNAs target 30 to 60% of human protein-coding genes [7, 8]. Numerous functions of microRNAs have been established both in negative regulation (transcriptional degradation or isolation, inhibition translation) and possible participation in positive regulation mechanisms (transcription and translation activation). MicroRNAs take part in the regulation of gene expression, so they are involved in most biological processes [9–11].

Abnormalities in miRNA expression have been shown in many diseases. The first disease in which a link with a microRNAs dysfunction was established was chronic lymphocytic leukemia [12]. Recent studies have shown the role of miR-205 in inhibiting the development of metastases in breast cancer [13]. In classical Hodgkin lymphoma, miR-21, miR-494, and miR-1973 present in blood plasma serve as markers of the disease [14]. MicroRNAs also play a regulatory role in the nervous system [15]. Neural microRNAs are involved in various stages of neural connection formation (miR-132, miR-134, and miR-124), synapse formation, and maturation [16].

miR-126 takes part in the development and progression of some autoimmune diseases, namely rheumatoid arthritis and systemic lupus erythematosus [17, 18]. Moreover, increased miR-126 expression enhances cell proliferation and reduces apoptosis in synovial fibroblasts in rheumatoid arthritis [17]. The level of miR-126 increases in the tissues of the large intestine during ulcerative colitis progression compared to values from the period between episodes and compared to the values of healthy people [19].

Data on miRNA dysregulation, including miR-31, miR-155, and miR-26b, is found in various immune diseases, particularly psoriasis [20–22]. When studying miR-126 in patients with psoriasis, Feng S *et al.* found that miR-126 expression in the lesions was increased compared to unaffected skin [23]. However, to our knowledge, no studies have been conducted on miR-126 expression in children with psoriasis. Also, depending on the miRNA levels in psoriasis, researchers are trying to develop and justify treatments. One study analyzed miRNA levels in psoriasis and proposed the use of a new biological therapy (anti-TNF-alpha) to reduce serum miRNA levels (including miR-126) in patients with psoriasis [34]. We aim to study the miR-126 expression level in children with psoriasis in the psoriatic and non-psoriatic epidermis, establish the impact on the characteristics of the course of psoriasis, and the results of therapy in children with psoriasis of initial expression levels of miR-126.

## Material and Methods

The clinical study involved 54 children with psoriasis aged 4 to 17 years, treated at the non-profit municipal enterprise (NPME) Kyiv City Clinical Dermatological and Venereological Hospital, in 2019 - early 2020. Inclusion criteria: (1) diagnosed with psoriasis, (2) children aged 4 to 17 years. Exclusion criteria: (1) age under 4 years, (2) age over 17 years, (3) children who were not regularly monitored.

Psoriasis was diagnosed based on clinical data and generally accepted diagnostic criteria. According to the latest recommendations, the severity of psoriasis in children was assessed by determining the Body surface (BSA), the total area of damage to the body. 1% of the body surface area is equal to the area of the patient’s palm: mild psoriasis was diagnosed with BSA<3 in 1 child, moderate with 3≤BSA≤10 in 15 children, and severe psoriasis with BSA>10 [24].

We have also calculated the Psoriasis Area and Severity Index (PASI) score, the index of prevalence and severity of psoriasis that reflects the area of the lesion, taking into account the intensity of signs such as erythema, peeling, and infiltration. The resulting score is 0 to 72 points. Moreover, we calculated the index I/PGA (Investigator/Physician’s Global Assessment Scale) or PGA – the index of severity of psoriasis, reflecting the intensity of the pathological process [25].

miR-126 extraction in individuals with psoriasis was carried out in the foci of psoriasis lesions and intact skin. Our study involved children with psoriasis, so the miR-126 extraction was carried out in the buccal epithelium and epithelium from the lesions: scales of psoriatic plaques and papules because taking a biopsy is an additional stress that can provoke an exacerbation of the pathological process. Buccal epithelium and epithelium from lesions collected with sterile brushes and scalpels were packed in sterile Eppendorf tubes. The material was stored at -20°C. The samples were transported to the laboratory under cold chain storage conditions.

Buccal epithelium and psoriatic epithelium were collected using sterile brushes, and scalpels were packed in sterile Eppendorf tubes. The material was stored at -20°C. The samples were transported to the laboratory under cold chain storage conditions.

The miRNA expression level was evaluated by real-time polymerase chain reaction (PCR). The TRIzol™ Reagent kit (Thermo Fisher Scientific, USA) was used to isolate total RNA and miRNA according to the provided instructions.

Total RNA concentration was determined using a NanoDrop ND1000 spectrophotometer (NanoDrop Technologies Inc. USA). The reverse transcription step was performed using the TaqMan® MicroRNA Reverse Transcription Kit (Applied Biosystems®, USA) and a loop-specific primer system for miR-126.

Real-time PCR results were detected using a 7500 Real-time PCR Systems amplifier by Applied Biosystems (Applied Biosystems, USA) using a mix of universal PCR Master Mix reagents (Applied Biosystems, USA). PCR was performed under the temperature regime: activation of AmpliTaq Gold DNA polymerase at 50°C for 2 minutes, primary denaturation of 95°C for 10 minutes, denaturation for 15 seconds at 60°C and another 60 seconds – primer annealing, elongation, and fluorescence detection, the last two steps were repeated for 40 cycles.

TaqMan® MIRNA Assays (hsa-miR-126) primers were used to determine the amount of miR-126 in the real-time PCR. miRNA expression levels were determined relative to U6B expression levels. miRNA levels were measured using the delta-Ct method.

Treatment of children with psoriasis was carried out using external therapy methods: topical glucocorticosteroids, topical vitamin D3 analogues or a combination of them, and phototherapy.

miR-126 expression levels from the buccal epithelium of 20 children who did not have chronic skin diseases – the control group – served as controls and compared the results.

The study materials were statistically processed using the StatTech v.1.2.0 software (developed by Stattech LLC, Russia). Quantitative parameters were evaluated for compliance with the normal distribution using the Shapiro-Wilk criterion (for less than 50 studies) and the Kolmogorov-Smirnov test (for more than 50 studies). Quantitative data with a normal distribution were described using arithmetic mean values (M) and standard deviation (SD) with a confidence interval limit (95% CI). In the absence of a normal distribution, we used the median (Me) and the lower and upper quartiles (Q1-Q3). Comparison of groups by quantitative parameters, the distribution of which differed from the normal one, was performed using the Mann-Whitney U test and the Kruskal-Wallis test. When comparing normally distributed quantitative indicators for two related samples, the Student’s paired t-test was used. To assess the diagnostic significance of quantitative signs in predicting a certain result, the ROC curve analysis was used. The value division of the quantitative feature at the cut-off point was determined by the highest value of the Youden index. The direction and strength of the correlation between quantitative indicators were estimated using Spearman’s rank correlation coefficient. The correlation coefficient value (r) was interpreted according to the Cheddock scale. A predictive model that characterized the dependence of a quantitative variable on factors represented by quantitative indicators was developed using the method of paired or multiple chain regression. Comparison of percentages in the analysis of multi-field connectivity tables was performed using the Pearson chi-square test (for values of the expected phenomenon greater than 10) and Fisher’s exact test (for values of the expected phenomenon less than 10). The odds ratio (OR) with a 95% confidence interval (CI) was used as a quantitative measure of the effect when comparing relative indicators.

## Results 

### Clinical and epidemiological characteristics of children with psoriasis

The median age of children with psoriasis was 12.07±0.44 years. There were 32 girls and 22 boys. According to the biological age periodization of children aged 4 to 11/12 years (children of both genders from 4 years – girls up to 11 years and boys up to 12 years), there were 25 children, and children aged 12/13 to 17 years (girls from 12 years and boys from 13 years to 17 years children of both sexes) – 29 children. The onset of the disease averaged 8.87±0.56 years. 19 (35.19%) of the 54 children were newly diagnosed with psoriasis. The duration of the disease ranged from a few weeks to 10 years, averaging 3.31±0.51 years. Exacerbations in some children coincided with the onset of the disease and lasted from 2 weeks to 2 years, averaging 8.35±1.21 weeks. Among children with psoriasis, 28 (51.85%) children had an exacerbation of the pathological process within 4 weeks, and 26 (48.15%) children – 5 or more weeks.

The clinical picture of the pathological process was mainly represented by plaque psoriasis – 38 (70.37%), guttate psoriasis was diagnosed in 8 (14.82%) children, inverse psoriasis – in 4 (7.41%) children, scalp psoriasis – in 3 (5.55%) children, and palmoplantar psoriasis – in 1 (1.85%) child. Such clinical forms of psoriasis caused the presence of a common pathological process in 46 (85.19%) children and a limited one in 8 (14.81%) children.

Assessment of the severity of psoriasis in children according to the BSA showed that in most children (41 (75.93%), the index was higher than 10, which provided an average BSA of 26.68±2.41, that indicates a severe course of psoriasis in children in terms of the psoriasis-affected body surface area.

The calculated PASI score averaged 11.75±1.35, which also assesses the course of psoriasis in children as severe, although the largest number of children, 31 children (57.41%), had a PASI score of up to 10; 23 children (42.59%) – with PASI>10. At the end of treatment, the average PASI score was 2.88±0.33, and in 16 children (29.63%), the effectiveness of treatment did not achieve PASI 75. The average PGA score of 3.05±0.11 assessed the intensity of the pathological process in psoriasis in children as predominantly severe. A detailed analysis showed that in 21 (38.89%) children, the PGA score was 4 – severe course; moderate course was in 15 children (27.78%), PGA score 3, respectively; 17 children (31.48%) had the intensity of the pathological process at the level of 2, and 1 child with PGA=1.

### miR-126 expression levels

miR-126 expression in the buccal epithelium of children with psoriasis was significantly lower than the miR-126 expression level in the buccal epithelium of healthy children. There was no significant statistical difference between the expression of miR-126 in psoriatic keratinocytes and miR-126 in the buccal epithelium in children with psoriasis (Table 1).

**Table 1.** miR-126 expression in the psoriatic epidermis and buccal epithelium depending on the clinical and epidemiological features of psoriasis.

**Arms by various criteria**		**miR-126 expression**		**Statistical significance**
		**Buccal epithelium**	**Psoriatic epidermis**	
		**Me** **Q1–Q3**	**Me** **Q1–Q3**	
**Children with psoriasis (n=54)**		0.46 [0.11–1.80]	0.53 [0.16–1.07]	p=0.097
**Healthy children (n=20)**		2.05 [0.66–5.52]	–	
**Statistical significance**		p<0.001 *	–	
**Gender**	boys (n=22)	0.47 [0.15–1.55]	0.58 [0.34–1.03]	p=0.617
	girls (n=32)	0.39 [0.09–2.00]	0.52 [0.12–1.12]	p=0.091
**Statistical significance**		p=0.747	p=0.211	
**Age of children**	4 to 11/12 years old (n=25)	0.31 [0.09–1.09]	0.46 [0.15–0.95]	p=0.439
	12/13 to 16/17 years old (n=29)	0.62 [0.14–2.05]	0.55 [0.16–1.07]	p=0.034 *
**Statistical significance**		p=0.197	p=0.724	
**Duration of exacerbation**	1 to 4 weeks (n=28)	0.31 [0.13–1.92]	0.46 [0.15–0.79]	p=0.240
	≥5 weeks (n=26)	0.47 [0.08–1.51]	0.66 [0.16–1.23]	p=0.253
**Statistical significance**		p=0.608	p=0.621	
**Clinical forms of psoriasis**	plaque (n=38)	0.49 [0.10–2.06]	0.66 [0.16–1.32]	p=0.058
	other (n=16)	0.19 [0.13–0.62]	0.43 [0.16–0.56]	p=0.859
**Statistical significance**		p=0.126	p=0.040 *	
**Episode of the disease**	onset (n=19)	0.30 [0.14–1.06]	0.61 [0.26–1.26]	p=0.729
	relapse (n=35)	0.54 [0.09–2.01]	0.46 [0.11–0.88]	p=0.026 *
**Statistical significance**		p=0.299	p=0.137	

* – score differences are statistically significant (p< 0.05)

### miR-126 expression level depending on the clinical and epidemiological features of psoriasis

When comparing the miR-126 expression levels in psoriatic keratinocytes and buccal epithelium between the groups of children, depending on gender, duration of exacerbation, no significant differences were found (Table 1). However, there were significant differences between miR-126 expression in the psoriatic epidermis and buccal epithelium in the group of children with psoriasis aged 12/13 to 17 years (p=0.034) and in the group of children with relapses of the disease (p=0.026). Depending on the clinical form of psoriasis, there were significant statistical differences between miR-126 expression levels in psoriatic keratinocytes in the group of children with plaque psoriasis and the group of children with other clinical forms of dermatosis (p=0.040).

### miR-126 expression level depending on psoriasis severity

There were no statistically significant differences between miR-126 expression levels in the psoriatic epidermis and between miR-126 expression levels in the buccal epithelium between groups of children, depending on the severity of psoriasis determined by the BSA, PGA, and PASI indices (Table 2). There is a statistically significant difference between miR-126 expression levels in the buccal epithelium and miR-126 in psoriatic keratinocytes in the group of children with a PGA score of 4 (p=0.041) and in the group with PASI<75 (p=0.016).

**Table 2.** miR-126 expression in the psoriatic epidermis and buccal epithelium depending on the severity of psoriasis.

**Arms by various criteria**		**miR-126 expression**		**Statistical significance**
		**Buccal epithelium**	**Psoriatic epidermis**	
		**Me** **Q1–**Q3	**Me** **Q1–Q3**	
**Children with psoriasis (n=54)**		0.46 [0.11–1.80]	0.53 [0.16–1.07]	p=0.097
**Healthy children (n=20)**		2.05 [0.66–5.52]	–	
**Statistical significance**		p<0.001 *	–	
**BSA**	≤10 (n=13)	0.47 [0.21–0.86]	0.41 [0.10–0.70]	p=0.697
	>10 (n=41)	0.46 [0.08–2.36]	0.56 [0.17–1.27]	p=0.116
**Statistical significance**		p=0.968	p=0.058	
**PGA**	1–2 (n=18)	0.31 [0.15–2.06]	0.51 [0.10–0.78]	p=0.344
	3 (n=16)	0.50 [0.21–1.32]	0.65 [0.20–1.23]	p=0.484
	4 (n=20)	0.48 [0.06–2.37]	0.44 [0.16–1.12]	p=0.041 *
**Statistical significance**		p=0.949	p=0.527	
**PASI**	≤10 (n=31)	0.47 [0.15–1.55]	0.56 [0.17–0.84]	p=0.189
	>10 (n=23)	0.39 [0.05–2.37]	0.44 [0.16–1.17]	p=0.457
**Statistical significance**		p=0.731	p=0.512	
**Treatment outcome**	PASI 75 (n=39)	0.31 [0.08–1.88]	0.52 [0.1–1.22]	p=0.632
	PASI <75 (n=15)	0.97 [0.15–1.81]	0.54 [0.36–0.82]	p=0.016 *
**Statistical significance**		p=0.081	p=0.605	

* – score differences are statistically significant (p< 0.05)

### Determination of the influence of miR-126 initial levels in children with psoriasis on therapy results

To elucidate the effect of miR-126 expression in the non-psoriatic epidermis – buccal epithelium – on the effectiveness of traditional therapy, we allocated children with psoriasis into two groups based on the miR-126 level in the buccal epithelium and compared treatment results. The cut-off point value was taken as the miR-126 boundary level, which corresponded to the highest value of the Youden index during ROC analysis when comparing miR-126 expression indicators from the buccal epithelium in children with psoriasis and children in the control group (AUC=0.776±0.048; 95%; CI: 0.681 – 0.871; p<0.001). The appearance of a patient with psoriasis was predicted at a value of 0.64 or lower (the sensitivity and specificity of the method was 90.0% and 61.1%, respectively). 2 groups of patients were formed: group 1: 35 children with miR-126≤0.64, and group 2: 19 children with miR-126>0.64.

**Figure 1.**
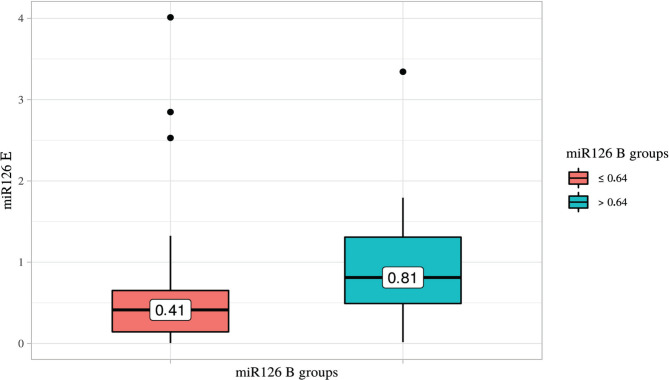
miR-126 expression level in keratinocytes across groups by miR-126 expression level in buccal epithelium (p=0.002).

The mean level of miR-126 expression in the buccal epithelium of children from Group 2 (2.36 [1.51–2.75]) was up to 15-fold higher than in Group 1 (0.15, [0.04–0.31], p<0.001). We found statistically significant differences between the groups when comparing the average miR-126 levels in psoriatic keratinocytes (p=0.002) (Figure 1).

With almost identical PASI scores at the beginning of treatment in both groups, at the end of treatment in Group 1, PASI is significantly statistically different from PASI in Group 2 (p=0.005) (Table 3).

**Table 3.** Dynamics of PASI in groups of children with psoriasis depending on miR-126 expression level in buccal epithelium.

**Groups** **by miR-126 level** **in buccal epithelium**	**Monitoring stages**				**Statistical significance**
	**PASI at the beginning of treatment**		**PASI at the end of treatment**		
	**Me**	**Q1–Q3**	**Me**	**Q1–Q3**	
**Group 1 (n=35)**	9.6	3.1–18.65	0	0–2.55	p<0.001 *
**Group 2 (n=19)**	8.4	4.3–15.3	1.45	0.70–2.23	p<0.001 *
**Statistical significance**	p=0.951		p=0.005 *		-

* – score differences are statistically significant (p< 0.05)

The percentage of children with psoriasis in Group 1 who did not achieve PASI75 during treatment was found to be 21.21%. Furthermore, in Group 2, the percentage of children with PASI<75 was almost 2-fold higher reaching 42.86%, which provided statistically significant differences between the groups of children by the number of successful treatments (PASI75) and treatment failures (PASI<75) (p=0.016). Based on the obtained data, the odds ratio showed that when treating children from Group 2, the chances of not reaching PASI75 at the end of therapy were 2.79-fold higher (OR 2.79; 95% CI: 1.19 – 6.51) compared with children of group 1.

## Discussion

Overgrowth of abnormal keratinocyte cells is the predominant sign of psoriasis, and excessive keratinocyte proliferation is closely associated with the occurrence of this disease. Keratinocytes in psoriasis initiate, maintain, and enhance pro-inflammatory responses by expressing molecules involved in attracting, retaining, and activating T cells [26, 27]. Inflammatory myeloid dendritic cells secrete IL-12 and IL-23, activate Th1 and Th22, producing IL-17, thereby increasing psoriatic inflammation [28].

However, the molecular mechanisms that precede these processes are not yet fully elucidated. As regulatory mechanisms, microRNAs play a significant role in diseases with an immune component, particularly psoriasis, preserving immune homeostasis, regulating innate and adaptive immunity, and pro-inflammatory factors.

One study of miR-126 in patients with psoriasis found increased miR-126 expression in psoriatic keratinocytes compared to intact skin and a positive correlation with PASI scores [23]. Another study found reduced plasma miR-126 levels in patients with psoriasis compared to patients in the control group (p<0.001) [29]. However, we also identified studies with no differences between miRNA-126 serum levels in patients with psoriasis and the control group [30].

In our study of children with psoriasis, miR-126 expression in the non-psoriatic epidermis (buccal epithelium) does not differ from miR-126 expression in psoriatic keratinocytes, although it is significantly lower than in children in the control group (AUC=0,776±0,048; 95%; CI: 0,681 – 0,871; p<0,001). Correlation analysis showed a direct moderate correlation between miR-126 expression in the buccal epithelium and miR-126 expression in affected keratinocytes in children with psoriasis (r=0.31; p=0.033), and if miR-126 expression in the buccal epithelium increases by 1.0, this will lead to an increase in miR-126 in psoriatic keratinocytes by 0.172. No other correlations were found between miR-126 expression levels and clinical features of the course and severity of psoriasis. However, it can be noted that miR-126 expression level in psoriatic keratinocytes determines the clinical form of psoriasis. This is confirmed by ROC analysis, which confirmed the statistical significance of the division into groups according to the clinical form of psoriasis (AUC=0.637±0.056; 95% CI: 0.528 – 0.747; p=0.014). It is possible to predict the course of plaque psoriasis with the value of the miR-126 expression in psoriatic keratinocytes equal to or greater than 0.66 (cut-off) (the sensitivity and specificity of the method was 51.4% and 84.6%, respectively). The same applies to moderate and severe psoriasis – with BSA>10. Although the expression of miR-126 in psoriatic keratinocytes at BSA>10 does not statistically differ from BSA≤10 when evaluating by ROC analysis the dependence of the probability of dividing children with psoriasis into groups according to the psoriasis-affected body surface area (BSA), a statistically significant model was obtained (AUC=0.634±0.063; 95% CI: 0.51 – 0.758; p=0.034). Moderate and severe psoriasis – BSA>10 – was predicted at miR-126=0.78 or higher (cut-off point) in psoriatic keratinocytes (the sensitivity and specificity of the method was 40.5% and 90.9%, respectively). Perhaps, this is because miR-126 promotes the proliferation of keratinocytes in psoriasis, which was also proved in a rat colitis model when miR-126 expression level was associated with the regulation of the inflammatory process [31].

In a study where miR-126 levels in the plasma of patients with psoriasis were lower than the control group, after 1, 3, and 6 months of treatment with TwHF + acitretin, miR-126 levels increased compared with initial indicators. Moreover, in patients who achieved PASI50 during treatment, the initial level of miR-126 in plasma was lower than in patients who did not achieve PASI50. According to the researchers, the initial high level of miR-126 was the reason for the failure in therapy [29]. In our study, the expression level of miR-126 in the buccal epithelium in children with psoriasis was also lower than in the control group, and the treatment efficiency of children with initial low expression levels of miR-126 in the buccal epithelium (up to 0.64) was higher than with high initial expression levels of miR-126 in the buccal epithelium. Failures in therapy with the expression of miR-126 in the buccal epithelium up to 0.64 were only in 21.21%, and when the expression level of miR-126 was higher than 0.64, they were almost two times higher – 42.86% (p=0.016).

On the one hand, Suresh Babu S. *et al.* found a low level of miR-126 expression in the cardiac tissue and the blood of diabetic patients compared with healthy individuals, which led to undesirable inflammatory reactions during wound healing and myocardial damage [32]. On the other hand, the Hu J. *et al.* study of tissue trauma suggested that high miR-126 expression in serum can suppress inflammation and facilitate wound healing after trauma by activating angiogenesis [33]. In what concerns psoriasis, the skin affected by the pathological process is characterized by the expansion of the superficial microcirculation vessels of the dermis, already changing at an early stage of psoriatic lesions development [35, 36]. Therefore, increased miR-126 expression in blood serum at psoriasis may induce endothelial cells to the expression of other factors, which, in turn, will lead to the progression of psoriasis [37] that will be reflected in engaging new skin areas to the pathological process. Moreover, the presence of increased miRNA levels in serum (including miR-126) in patients with psoriasis is undesirable prognostic evidence [34].

This was once again confirmed when calculating the composition of groups based on the level of successful therapy in children, where there are statistically significant differences between miR-126 levels in keratinocytes and buccal epithelium by age and PGA severity index. In the 12/13-17-year-old group, the number of children (37.9%) who did not reach PASI75 during treatment is almost 2 times bigger than the group of 4-11/12-year-old children (20.0%; p=0.042). The chance of not achieving a positive result in treatment in the 12/13-17-year-old children group is 2.44 times higher than in the group of 4-11/12-year-old children (OR 2.44; 95% CI: 1.02 – 5.85). The largest percentage of children (45%) who were treatment failures (depending on the severity of the pathological process in terms of intensity levels of skin manifestations (PGA)) were children with PGA=4, which significantly differed from the group with PGA=1-2 (27,8%; p=0.011) and the group with PGA=3 (12,5%; p=0.02). Furthermore, the chances of not achieving positive treatment results in children with PGA=4 are 3.16 times higher than those with lower severity of psoriasis according to PGA (OR 3.16; 95% CI: 1.34 – 7.43).

## Conclusions

MicroRNAs play a significant role in the pathogenesis of psoriasis; these studies are ambiguous and need to be improved. The results of our study showed that in children with psoriasis, the levels of miR-126 expression in the buccal epithelium do not differ from the levels of miR-126 expression in psoriatic keratinocytes but are significantly lower than the expression of miR-126 in the buccal epithelium of healthy children. Given that the place of miR-126 expression determines the effect on the course of psoriasis and treatment outcome, the high level of miR-126 in keratinocytes affected by psoriasis leads to manifestations of plaque psoriasis with a course in moderate to severe and severe forms according to the psoriasis-affected body surface area. Initial high miR-126 levels in the buccal epithelium in children with psoriasis are a negative prognostic criterion for response to therapy and can be used as a marker for prescribing systemic treatment to children with psoriasis.

## Acknowledgments

### Conflict of interest

The authors declare that there is no conflict of interest.

### Ethics approval

The study was approved by the Ethics Commission of Shupyk National Healthcare University of Ukraine (No.4, 03/02/2020).

### Consent to participate

All participants received and signed informed consents before entering the study.

### Authorship

DV and LO made the research concept, ME, DE designed the study, ME, VS conducted the literature review, ME, BK handled collection and processing of materials, DT, DV conducted the laboratory research, ME conducted the statistical processing of the material, ME, VS, BK wrote the article, and DV, LO edited the article.
